# Availability of essential medicines in primary health care of the Brazilian Unified Health System

**DOI:** 10.11606/S1518-8787.2017051007062

**Published:** 2017-09-22

**Authors:** Renata Cristina Rezende Macedo do Nascimento, Juliana Álvares, Augusto Afonso Guerra, Isabel Cristina Gomes, Ediná Alves Costa, Silvana Nair Leite, Karen Sarmento Costa, Orlando Mario Soeiro, Ione Aquemi Guibu, Margô Gomes de Oliveira Karnikowski, Francisco de Assis Acurcio

**Affiliations:** I Programa de Pós-Graduação em Medicamentos e Assistência Farmacêutica. Faculdade de Farmácia . Universidade Federal de Minas Gerais . Belo Horizonte , MG , Brasil; II Departamento de Farmácia Social . Faculdade de Farmácia . Universidade Federal de Minas Gerais . Belo Horizonte , MG , Brasil; III Faculdade de Ciências Médicas de Minas Gerais . Belo Horizonte , MG , Brasil; IV Instituto de Saúde Coletiva . Universidade Federal da Bahia . Salvador , BA , Brasil; V Departamento de Ciências Farmacêuticas . Universidade Federal de Santa Catarina . Florianópolis , SC , Brasil; VI Núcleo de Estudos de Políticas Públicas . Universidade Estadual de Campinas . Campinas , SP , Brasil; VII Programa de Pós-Graduação em Saúde Coletiva. Departamento de Saúde Coletiva . Faculdade de Ciências Médicas . Universidade Estadual de Campinas . Campinas , SP , Brasil; VIII Programa de Pós-Graduação em Epidemiologia. Faculdade de Medicina . Universidade Federal do Rio Grande do Sul . Porto Alegre , RS , Brasil; IX Faculdade de Ciências Farmacêuticas . Pontifícia Universidade Católica de Campinas . Campinas , SP , Brasil; X Departamento de Saúde Coletiva . Faculdade de Ciências Médicas . Santa Casa de São Paulo . São Paulo , SP , Brasil; XI Faculdade de Ceilândia . Universidade de Brasília . Brasília , DF , Brasil

**Keywords:** Drugs, Essential, supply & distribution, Pharmaceutical Services, Primary Health Care, Health Services Research, Unified Health System, Medicamentos Essenciais, provisão & distribuição, Assistência Farmacêutica, Atenção Primária à Saúde, Pesquisa sobre Serviços de Saúde, Sistema Único de Saúde

## Abstract

**OBJECTIVE:**

To characterize the availability of tracer medicines in pharmaceutical services in primary health care of the Brazilian Unified Health System (SUS).

**METHODS:**

This is a cross-sectional and evaluative study, part of the *Pesquisa Nacional Sobre Acesso, Utilização e Promoção do Uso Racional de Medicamentos* – *Serviços, 2015* (PNAUM – National Survey on Access, Use and Promotion of Rational Use of Medicines – Services, 2015). To analyze the availability of medicines, we verified 50 items selected from the *Relação Nacional de Medicamentos Essenciais* (Rename – National List of Essential Medicines) of 2012. Observation scripts were applied to medicine dispensing services in the primary health care. Interviews were carried out with patients, health care professionals, and public managers, using semi-structured questionnaires. The availability index was presented as the percentage of health units where the medicines were available. For statistical analysis, absolute, relative, and mean frequencies were presented (with 95% confidence intervals). The comparison of groups was carried out by Pearson Chi-square tests or variance analysis, when needed.

**RESULTS:**

One thousand, one hundred, and seventy-five observation scripts were filled in a national representative sample composed by 273 cities. Statistically significant differences were observed regarding the type of unit, infrastructure, and presence of a pharmacist between regions of Brazil. The average availability of tracer medicines in primary health care was 52.9%, with differences between regions and sampling strata. This index increased to 62.5% when phytotherapic medicines were excluded. We found limited availability of medicines for treatment of chronic and epidemiological diseases, such as tuberculosis and congenital syphilis.

**CONCLUSIONS:**

The low availability of essential medicines purchased centrally by the Brazilian Ministry of Health indicates deficiencies in supply chain management. The different views on the availability of tracer medicines in SUS confirm the general availability verified in this study. Among patients, about 60% said they obtain medicines in SUS units, data consistent with the lack of medicines reported by medicine dispensers and in line with physicians’ evaluations.

## RESUMO

**OBJETIVO:**

Caracterizar a disponibilidade física de medicamentos traçadores nos serviços de assistência farmacêutica na atenção primária do Sistema Único de Saúde.

**MÉTODOS:**

Estudo transversal de natureza avaliativa, integrante da Pesquisa Nacional Sobre Acesso, Utilização e Promoção do Uso Racional de Medicamentos – Serviços, 2015. Para a análise da disponibilidade física, foram verificados 50 itens selecionados da Relação Nacional de Medicamentos Essenciais 2012. Roteiros de observação foram aplicados nos serviços de dispensação de medicamentos na atenção primária. Foram realizadas entrevistas com usuários, profissionais de saúde e gestores municipais, por meio de questionários semiestruturados. O índice de disponibilidade foi apresentado como o percentual de unidades de saúde onde os medicamentos estavam disponíveis. Para a análise estatística foram apresentadas frequências absolutas, relativas e médias (com intervalos de 95% de confiança). A comparação de grupos foi realizada por meio dos testes Qui-quadrado de Pearson ou análise de variância, quando adequados.

**RESULTADOS:**

Foram preenchidos 1.175 roteiros de observação em amostra nacional representativa composta por 273 municípios. Observaram-se diferenças estatisticamente significantes em relação ao tipo de unidade, infraestrutura e presença do profissional farmacêutico entre as regiões do Brasil. A disponibilidade média dos medicamentos traçadores na atenção primária foi de 52,9%, com diferenças entre regiões e estratos amostrais. Quando analisados todos os medicamentos, exceto os fitoterápicos, o índice elevou para 62,5%. Verificou-se disponibilidade inadequada de medicamentos para o tratamento de doenças crônicas e para doenças epidemiologicamente importantes, como a tuberculose e a sífilis congênita.

**CONCLUSÕES:**

A baixa disponibilidade de medicamentos de aquisição centralizada indica possíveis deficiências na gestão da cadeia logística. As diferentes percepções sobre a disponibilidade dos medicamentos traçadores no SUS corroboram com os índices de disponibilidade geral verificados pelo estudo. Dentre os usuários, aproximadamente 60% afirmaram obter os medicamentos que necessitaram nas unidades do SUS, informação coerente com a falta de medicamentos relatada pelos responsáveis pela dispensação de medicamentos e com a avaliação dos médicos.

## INTRODUCTION

Primary health care is characterized by individual and collective actions, comprising health promotion and protection, injury prevention, diagnosis, treatment, rehabilitation, and health maintenance. High-complexity and low-density technologies are used to solve relevant problems in the health services’ territory. Medicines are one of the most important technologies used by modern society and a fundamental health care therapeutic resource ^21^ ^,^ ^23^ .

Important changes in the epidemiological profile that occurred in the last century led to a significant increase in life expectancy and increase in life expectancy and predominance of predominance of chronic non-communicable diseases (CNCD) ^3^ ^,^ ^12^ . Currently, health care in Brazil is characterized by an accelerated demographic transition and expressed by triple burden of disease: an agenda of infectious diseases not yet overcome, diseases associated with external causes, and the chronic conditions’ hegemony. The answer to this situation has been structured by the health care network, with access to actions and services coordinated by primary health care ^8^ ^,^ ^12^ .

In this context, pharmaceutical services play an essential role. One of the nine global goals for CNCD control is to ensure 80% of availability of essential medicines and basic technologies, since the appropriate pharmacological treatment significantly reduces morbidity and mortality of these diseases. Appropriate availability, associated with sustainable funding and improvement of the health care system, contribute to ensure a universal coverage ^3^ ^,^ ^7^ ^,^ ^24^ ^,^ ^25^ .

Pharmaceutical services in primary health care aim to ensure a comprehensive, continuous, and compatible health care for the population, with medicines being one of the essential elements ^21^ . As part of the constitutional right to health, the pharmaceutical services in the Brazilian Unified Health System (SUS) ^a^ comprise administrative activities to ensure an adequate availability of medicines, their quality and conservation, besides assistance services, focusing on therapeutic effectiveness and safety ^21^ .

Ensuring that the population has access to essential medicines is one of the eight Millennium Development Goals ^26^ and, therefore, one pillar of the national health care policies formulation and implementation ^20^ . Despite being a fundamental factor of the right to health, the access to essential medicines to prevent and treat high prevalence disorders, including chronic respiratory and cardiovascular diseases and diabetes, it may still be considered low and irregular worldwide ^2^ ^,^ ^8^ ^,^ ^21^ .

The concept of access is wide and comprises five dimensions – availability, accessibility, accommodation, acceptability, and affordability ^10^ ^,^ ^22^ . Availability is an important and challenging aspect, especially in publicly funded universal health systems. It is defined as the relationship between the type and quantity of products required and those offered ^11^ ^,^ ^20^ . Under the perspective of public and universal health care systems, the analysis of availability is often taken as a proxy for the evaluation of the access to essential medicines.

Studies carried out at Brazilian primary health care units point out issues on the availability and quality of drugs use and deficiencies in logistics and supply chain management, despite the legal framework of pharmaceutical services ^a^ and growing investments made by the three levels of SUS management ^3^ ^,^ ^9^ ^,^ ^13^ ^,^ ^20^ . The *Pesquisa Nacional sobre Acesso, Utilização e Promoção do Uso Racional de Medicamentos* – *Serviços* (PNAUM – National Survey on Access, Use and Promotion of Rational Use of Medicines – Services) aimed to characterize pharmaceutical services in SUS primary health care – for promoting the access and rational use of medicines –, as well as to identify and discuss the factors that affect the consolidation of pharmaceutical services in the municipalities.

This study aimed at identifying factors related to the availability of tracer medicines in pharmaceutical services of SUS primary health care.

## METHODS

This study is part of PNAUM – Services, a cross-sectional, exploratory, and evaluative survey, with a representative sample of cities, primary health care services, patients, physicians, and medicine dispensers in the five regions of Brazil. The sampling plan considered the different populations under study and estimated different sampling sizes for each population ^1^ . In each region, 120 cities, 300 health care services, and 1,800 users were sampled. The total sample (600 cities) was stratified in capitals (26 and the Federal District); biggest cities (the 0.5% biggest cities in the region, totaling 27), and smallest cities in population (546 cities chosen by lot). For obtaining the health care services’ sample, sixty cities by region were chosen by lot among the 120 previously selected. Primary health care units, health care centers, or basic/mixed health units were included in the draw of health services, according to the *Cadastro Nacional de Estabelecimentos de Saúde* (CNES – National Register of Health Establishments). Face-to-face interviews were held with patients, physicians, and medicine dispensers in primary health care services, as well as telephone interviews with those responsible for pharmaceutical services in the cities, using a structured questionnaire specific for each category. Respondents were asked about availability of medicines in the three-month period before the interview. Pharmaceutical services’ facilities and availability of medicines were assessed using an observation script. A handbook and a glossary of technical terms were elaborated for each survey instrument. After training the interviewers, a pretest was carried out, involving cities with different population size to improve and validate the instruments. The data were collected between July 2014 and May 2015.

Availability was assessed using a list of tracer medicines. A group of experts has selected 58 medicines from the *Relação Nacional de Medicamentos Essenciais* (Rename – National List of Essential Medicines) of 2012, grouped according to their therapeutic class in 50 assessment items. The guiding criterion for choosing them were the parameters established by the World Health Organization (WHO) ^25^ to enable researches on availability of essential medicines ^b^ , including those indicated for the treatment of epidemiological relevant conditions in the Country within primary health care.

Availability was defined as the presence of at least one pharmaceutical unit of the medicine, visible at the time of data collection, according to direct observation of the field researcher. For medicines in the same therapeutic category, grouped in one item, we considered the availability of at least one unit among therapeutic options established. The availability index was presented as the percentage of health units evaluated where the medicines were available. For statistical analysis, absolute, relative, and mean frequencies were presented (with 95%CI for relative and mean frequencies). The comparison of groups was made using Pearson Chi-square tests or variance analysis, when needed.

Data analysis was performed using the software SPSS® version 22, using the plan of complex samples ^1^ . The research was approved by the National Research Ethics Committee, under CONEP opinion no. 398.131/2013. All interviews were preceded by explanation of the research objectives to the respondent and by the signing of the informed consent form.

## RESULTS

In total, 1,175 observation scripts were filled in 273 Brazilian cities. For organizational issues of pharmaceutical services, some of the cities centralize the dispensing of medicines in public pharmacies located in independent buildings or in bigger health centers. Statistically significant differences were observed between Brazilian regions, regarding type of unit, infrastructure, and existence of pharmacist in units that supply medicines where the observation script were applied ( Table 1 ).

**Table 1:** Description of medicine dispensing units in SUS primary health care. National Survey on Access, Use and Promotion of Rational Use of Medicines – Services, 2015.

Variable	North		Northeast		Midwest		Southeast		South		Brazil	
					
	n ^a^	% (IC95%)	n ^a^	% (IC95%)	n ^a^	% (IC95%)	n ^a^	% (IC95%)	n ^a^	% (IC95%)	n ^a^	% (IC95%)
Type of unit ^b^												
Center/basic health unit	179	68.8 (62.3–74.6)	181	72.9 (63.9–80.3)	53	25.4 (16.2–37.5)	124	42.0 (29.7–55.5)	171	64.5 (52.1–75.1)	708	58.8 (53–64.3)
Health Center	24	8.7 (5.7–13.1)	56	13.9 (9.8–19.4)	88	28.4 (18.7–40.6)	49	15.4 (10.4–22.4)	40	12.4 (7.6–19.7)	257	14.6 (12–17.6)
Pharmacy in an independent building	37	15.5 (11.2–20.9)	6	4.1 (1.0–15.3)	20	37.4 (20.2–58.5)	22	17.8 (10.4–28.8)	31	16.8 (7.9–32.4)	116	13.4 (9.6–18.3)
Mixed unit	8	3.3 (1.6–6.5)	11	4.3 (1.7–10.5)	16	7.5 (3.1–16.8)	31	20.3 (9.9–37.3)	7	5.7 (1.9–15.7)	73	9.6 (5.7–15.8)
Other	11	3.8 (2.0–7.1)	5	4.8 (1.6–13.5)	1	1.3 (0.2–9.0)	3	4.4 (0.8–20.5)	1	0.6 (0.1–4.0)	21	3.7 (1.6–8.2)
Unit has a responsible pharmacist ^b^	78	26.8 (21.9–32.2)	52	18.5 (11.7–28.1)	95	66.9 (55.6–76.6)	176	72.0 (57.3–83.1)	105	44.8 (34.5–55.6)	506	43.0 (37.8–48.4)
Equipment and furnishings of the storage area ^b^												
Exclusive refrigerator for medicines	97	37.2 (31.0–43.8)	97	21.3 (16.1–27.6)	112	50.7 (36.6–64.7)	180	76.0 (65.9–83.9)	164	56.8 (46.7–66.4)	650	47.2 (42.0–52.5)
Air conditioner	163	59.0 (52.4–65.4)	92	21.3 (14.8–29.5)	103	72.7 (61.6–81.6)	101	46.0 (33.1–59.4)	76	37.1 (26.7–48.8)	535	37.7 (32.4–43.2)
Locker cabinet ^c^	99	38.2 (32.0–44.7)	66	22.6 (15.9–31.1)	80	65.1 (50.7–77.3)	154	63.4 (49.6–75.3)	103	48.9 (38.5–59.5)	502	43.4 (38.0–49.0)

^a^ Non-weighted n value

^b^ p-value < 0.05

^c^ For storage of medicines subject to special control, such as antipsychotics, anxiolytics, and sedatives, regulated by Ordinance GM/MS no. 344/98.

Source: PNAUM – Services, 2015.

The average availability of tracer medicines in SUS primary health care was 52.9%, with statistically significant differences between regions (p<0.05). We observed relevant amplitude between the evaluated items ( Table 2 ). Oral re-hydration salts presented the highest availability (91.9%) and phytotherapic medicines, the lowest (ranging from 0.8% to 8.6%). When we analyzed all medicines, except phytotherapic ones, the average increased to 62.5%.

**Table 2:** Average availability of essential medicines in the visited primary health care dispensing units. National Survey on Access, Use and Promotion of Rational Use of Medicines – Services, 2015.

Medicine	Availability % (95%CI)					

	North	Northeast	Midwest	Southeast	South	Brazil
Anti-hypertensives	73.5 (68.9–78.2)	85.9 (82.2–89.6)	86.1 (80.5–91.6)	91.8 (88.5–95.1)	84.0 (78.7–89.4)	84.3 (82.2–86.3)
1. Captopril/enalapril ^a^	84.1 (78.5–88.4)	84.4 (78.3–89.0)	84.1 (75.9–89.9)	95.4 (91.2–97.6)	83.9 (76.7–89.3)	87.7 (84.8–90.1)
2. Hydrochlorothiazide	72.9 (66.5–78.5)	86.6 (80.1–91.2)	81.7 (73.2–88.0)	86.1 (79.6–90.8)	83.5 (76.1–89.0)	84.5 (81.3–87.3)
3. Atenolol/propranolol/carvedilol/metoprolol ^a^	70.7 (64.2–76.4)	66.2 (58.4–73.1)	77.6 (65.1–86.6)	90.9 (85.0–94.7)	79.2 (69.8–86.2)	77.1 (73.1–80.6)
Oral antidiabetics and insulin	66.4 (61.7–71.1)	77.1 (72.9–81.3)	80.5 (72.6–88.4)	91.1 (87.7–94.4)	74.9 (69.0–80.8)	78.0 (75.6–80.4)
4. Metformin ^a^	79.2 (73.4–84.0)	86.4 (80.0–90.9)	83.8 (74.8–90.1)	91.8 (84.9–95.7)	74.0 (61.4–83.5)	85.2 (81.4–88.4)
5. Glibenclamide/glicazide	79.2 (73.1–84.2)	85.3 (76.8–91.1)	84.3 (74.3–90.9)	84.6 (76.9–90.0)	72.9 (64.3–80.1)	82.4 (78.5–85.8)
6. NPH human insulin ^a^	49.5 (42.9–56.0)	55.5 (47.3–63.4)	65.6 (53.0–76.4)	90.3 (84.5–94.1)	67.8 (57.9–76.3)	68.4 (64.0–72.6)
7. Regular human insulin ^a^	45.2 (38.7–51.8)	50.6 (42.4–58.8)	64.9 (52.2–75.9)	84.6 (77.3–89.9)	59.8 (49.8–69.0)	63.1 (58.2–67.7)
Contraceptives/hormones	56.3 (51.2–61.4)	69.5 (63.2–75.7)	81.8 (76.0–87.5)	88.3 (83.8–92.9)	83.1 (78.7–87.5)	75.8 (73.5–78.1)
8. Ethinylestradiol + levonorgestrel ^a^	48.6 (42.3–54.9)	63.1 (54.6–70.8)	84.1 (75.6–90.1)	87.0 (80.0–91.8)	86.6 (79.1–91.7)	74.5 (70.1–78.4)
9. Norethindrone + estradiol ^a^	53.1 (46.6–59.4)	64.1 (55.7–71.8)	78.6 (66.4–87.2)	82.7 (74.7–88.6)	87.6 (80.8–92.2)	73.8 (69.3–77.8)
10. Norethisteronea	52.0 (45.6–58.4)	65.5 (57.1–73.1)	72.9 (61.6–81.8)	73.8 (58.8–84.8)	82.3 (75.3–87.6)	70.2 (64.7–75.2)
11. Medroxyprogesterone ^a^	44.9 (38.7–51.4)	45.1 (37.1–53.3)	61.7 (50.1–72.1)	71.6 (61.1–80.3)	72.1 (63.3–79.5)	58.8 (53.6–63.8)
12. Levonorgestrel	46.3 (40.1–52.7)	54.1 (45.7–62.3)	66.5 (56.3–75.4)	62.5 (46.7–76.1)	62.1 (52.3–70.9)	58.2 (52.2–63.8)
13. Estriol vaginal cream ^a^	15.4 (11.5–20.3)	20.0 (14.8–26.4)	38.7 (28.3–50.3)	15.4 (10.9–21.3)	27.9 (20.0–37.4)	20.6 (17.5–24.2)
14. Conjugated Estrogens Vaginal cream	10.2 (7.0–14.4)	17.7 (12.4–24.7)	31.0 (20.0–44.7)	18.3 (12.4–26.2)	20.8 (14.5–28.9)	18.6 (15.3–22.4)
Anti-infectious	62.7 (58.2–67.2)	70.0 (65.4–74.7)	76.7 (70.5–82.8)	83.9 (80.1–87.8)	73.3 (66.7–79.9)	73.3 (70.9–75.7)
15. Fluconazole/itraconazol ^a^	69.2 (62.6–75.0)	67.2 (59.3–74.3)	83.2 (76.8–88.0)	88.5 (82.1–92.8)	67.3 (57.8–75.6)	74.9 (70.8–78.7)
16. Miconazole nitrate ^a^	62.7 (56.0–68.9)	64.1 (56.1–71.4)	72.4 (63.1–80.1)	83.0 (74.9–88.9)	84.4 (75.2–90.6)	73.7 (69.4–77.6)
17. Ciprofloxacin chloride ^a^	51.4 (44.8–57.8)	59.7 (51.6–67.3)	60.7 (49.4–70.9)	82.7 (74.5–88.7)	54.0 (43.8–63.9)	65.3 (60.5–69.7)
18. Nystatin cream ^a^	44.0 (37.6–50.6)	54.7 (46.6–62.6)	60.1 (44.2–74.0)	68.0 (57.0–77.3)	53.1 (42.4–63.5)	58.0 (52.7–63.1)
19. Benzathine benzylpenicilin ^a^	59.0 (52.5–65.3)	37.2 (29.7–45.4)	49.0 (37.4–60.8)	56.3 (42.0–69.7)	60.7 (50.9–69.7)	49.5 (44.0–55.0)
Analgesics/antipyretics/anti-inflammatory	73.9 (69.2–78.6)	80.6 (75.8–85.5)	85.9 (78.8–93.0)	91.0 (86.3–95.7)	95.3 (92.5–98.0)	85.3 (83.1–87.6)
20. Paracetamol ^a^	84.8 (79.4–89.1)	83.4 (77.2–88.2)	87.2 (77.6–93.1)	95.4 (90.5–97.8)	98.9 (95.9–99.7)	90.1 (87.3–92.3)
21. Dipyrone oral solution	86.0 (80.6–90.1)	83.0 (76.9–87.7)	83.3 (73.0–90.2)	86.2 (74.4–93.1)	93.5 (87.3–96.7)	86.0 (82.0–89.3)
22. Ibuprofen ^a^	58.8 (52.2–65.1)	57.4 (49.1–65.4)	83.4 (73.7–90.0)	75.3 (58.8–86.8)	94.3 (89.7–96.9)	70.8 (65.1–75.9)
Antiemetics/antisecretory	54.3 (49.5–59.1)	65.3 (59.6–71.0)	76.5 (68.3–84.7)	80.3 (73.1–87.5)	83.2 (75.9–90.4)	71.9 (68.9–74.9)
23. Omeprazol ^a^	32.0 (26.3–38.4)	55.1 (46.7–63.1)	54.6 (41.6–66.9)	82.8 (73.8–89.1)	85.9 (76.7–91.9)	66.9 (62.1–71.4)
24. Aluminium hydroxide ^a^	64.7 (58.5–70.5)	64.4 (56.0–72.0)	77.5 (69.7–83.8)	44.4 (32.4–57.1)	68.4 (55.7–78.8)	59.7 (53.7–65.5)
25. Ranitidine hydrochloride	59.6 (52.8–66.0)	50.2 (42.0–58.4)	70.0 (59.4–78.8)	61.3 (46.9–74.0)	59.7 (48.1–70.2)	57.1 (51.4–62.7)
Anti-asthmatics	67.4 (62.3–72.5)	78.2 (73.0–83.5)	82.1 (76.0–88.1)	88.8 (84.4–93.2)	83.6 (78.7–88.5)	80.0 (77.7–82.3)
26. Prednisolone sodium phosphate/prednisolone ^a^	64.9 (58.3–71.0)	74.6 (65.7–81.7)	83.6 (76.8–88.7)	86.6 (75.6–93.1)	80.5 (66.1–89.9)	79.0 (74.0–83.3)
27. Salbutamol sulphate ^a^	61.6 (55.0–67.8)	59.3 (50.9–67.2)	69.2 (55.9–79.9)	81.3 (70.6–88.7)	64.2 (54.8–72.6)	67.7 (62.7–72.3)
28. Ipratropium bromide	57.3 (50.6–63.7)	57.9 (49.8–65.7)	62.1 (50.5–72.5)	60.5 (46.4–73.0)	70.8 (60.9–79.1)	61.1 (55.5–66.4)
Antiparasitic agents	70.3 (65.5–75.1)	84.2 (77.9–90.5)	83.1 (76.5–89.7)	91.2 (87.5–94.8)	85.7 (80.1–91.3)	82.9 (80.4–85.4)
29. Albendazol ^a^	87.9 (83.0–91.6)	81.4 (72.4–87.9)	80.7 (67.8–89.2)	92.3 (87.3–95.4)	95.9 (92.4–97.8)	87.7 (83.8–90.7)
30. Metronidazole/teclozan ^a^	74.0 (68.1–79.1)	82.4 (73.8–88.6)	77.4 (64.2–86.8)	89.3 (83.4–93.3)	78.1 (69.8–84.7)	82.9 (78.9–86.2)
31. Permethrin ^a^	15.5 (11.6–20.4)	46.6 (38.7–54.7)	55.7 (46.1–64.9)	57.5 (43.7–70.2)	80.0 (71.9–86.1)	53.6 (48.1–59.0)
Psychotropics	15.0 (11.0–19.0)	10.6 (4.4–16.9)	41.6 (30.2–53.0)	48.0 (35.0–61.1)	45.7 (35.0–56.4)	32.2 (27.9–36.5)
32. Amitriptyline hydrochloride ^a^	17.7 (13.5–22.8)	10.8 (5.9–19.0)	38.4 (27.1–51.1)	49.3 (36.3–62.5)	50.4 (40.2–60.6)	31.5 (26.4–37.1)
33. Carbamazepine ^a^	17.4 (13.3–22.5)	12.5 (7.0–21.3)	42.1 (29.9–55.2)	46.0 (33.2–59.4)	47.3 (36.8–580.)	30.8 (25.6–36.6)
34. Fluoxetine ^a^	11.2 (8.0–15.4)	8.6 (4.1–17.3)	34.8 (22.2–49.9)	50.3 (37.1–63.4)	48.3 (37.9–58.8)	29.9 (24.8–35.6)
35. Clonazepam ^a^	17.9 (13.8–22.9)	8.8 (4.3–17.1)	36.2 (24.2–50.2)	46.1 (33.3–59.4)	34.0 (23.2–46.8)	26.9 (21.9–32.6)
Tuberculostatics	32.5 (27.7–38.2)	10.6 (5.9–15.4)	32.2 (21.3–43.2)	44.7 (31.3–58.1)	9.9 (6.4–13.3)	26.0 (22.2–29.8)
36. Isoniazid 75 mg + rifampicin 150 mg + pyrazinamide 400 mg + ethambutol 275 mg ^a^	35.6 (29.7–42.0)	10.2 (6.4–15.9)	31.8 (22.0–43.5)	40.7 (29.3–53.3)	9.4 (6.4–13.4)	22.9 (19.1–27.1)
37. Rifampicin 300 mg ^a^	30.4 (24.8–36.7)	9.4 (5.6–15.4)	27.8 (18.1–40.1)	36.7 (24.2–51.4)	7.1 (4.7–10.6)	20.3 (15.6–26.0)
Phytotherapics	9.8 (7.4–12.2)	2.8 (0.7–4.9)	12.4 (0.5–24.3)	13.9 (7.5–20.4)	10.8 (6.0–15.6)	8.8 (6.4–11.0)
38. Guaco ^a^	10.6 (8.4–13.4)	2.7 (1.2–5.8)	12.0 (4.2–29.7)	12.7 (8.4–18.9)	11.5 (7.2–17.8)	8.5 (6.6–10.7)
39. Soy isoflavone ^a^	1.5 (0.8–3.0)	0.3 (0.1–1.3)	7.4 (1.2–33.6)	10.0 (6.1–15.9)	3.8 (1.7–8.4)	4.4 (3.0–6.4)
40. Unha-de-gato ^a^	–	–	–	9.5 (5.8–15.3)	–	3.0 (1.9–4.5)
41. Espinheira-santa ^a^	1.2 (0.5–2.6)	0.2 (0.0–1.1)	7.2 (1.2–33.9)	1.8 (0.9–3.6)	2.6 (1.0–6.9)	1.6 (0.8–3.0)
42. Aroeira ^a^	–	0.5 (0.2–1.4)	7.2 (1.2–33.9)	1.2 (0.3–4.2)	–	1.0 (0.4–2.6)
43. Cáscara-sagrada ^a^	0.2 (0.0–1.4)	0.2 (0.0–1.1)	0.5 (0.1–3.6)	2.9 (0.7–12.0)	–	1.0 (0.3–3.7)
44. Garra-do-diabo ^a^	0.2 (0.0–1.2)	–	0.1 (0.0–0.9)	2.0 (1.0–3.8)	1.0 (0.2–3.7)	0.8 (0.5–1.4)
45. Artichoke	2.0 (1.1–3.5)	0.6 (0.3–1.6)	–	1.4 (0.6–3.3)	–	0.8 (0.5–1.4)
Other medicines	70.6 (65.8–75.5)	81.4 (76.3–86.6)	81.3 (75.3–87.3)	92.3 (88.7–95.8)	79.5 (74.4–84.5)	81.0 (78.8–83.2)
46. Oral rehydration salts	88.0 (83.0–91.7)	89.7 (83.5–93.7)	89.5 (82.1–94.1)	95.8 (90.2–98.3)	92.3 (87.2–95.5)	91.9 (89.1–94.0)
47. Ferrous sulfate ^a^	69.6 (63.3–75.3)	90.4 (85.1–93.9)	84.8 (75.6–91.0)	94.6 (90.2–97.1)	91.5 (82.9–96.0)	89.9 (87.2–92.0)
48. Dexamethasone cream/ointment ^a^	70.0 (63.6–75.8)	69.1 (61.2–76.1)	82.7 (74.5–88.6)	90.7 (82.4–95.3)	86.0 (76.1–92.2)	79.5 (75.3–83.1)
49. Folic acid ^a^	65.5 (58.9–71.6)	72.9 (65.2–79.4)	72.4 (58.3–83.2)	81.0 (70.9–88.1)	87.8 (80.3–92.7)	77.3 (72.9–81.1)
50. Nicotine ^a^	4.5 (2.4–8.1)	4.4 (2.0–9.3)	34.0 (23.0–46.9)	27.2 (17.8–39.3)	17.0 (8.5–31.1)	15.3 (11.6–19.9)

General availability	44.6 (42.8–46.3)	46.3 (44.2–48.4)	55.9 (51.3–60.5)	60.5 (58.3–62.8)	56.8 (54.6–58.9)	52.9 (51.6–54.2)
General availability excluding phytotherapics ^b^	52.7 (50.6–54.8)	55.0 (52.5–57.5)	65.7 (60.9–70.5)	71.1 (68.6–73.6)	67.2 (64.6–69.7)	62.5 (60.9–64.0)

NPH: Neutral Protamine Hagedorn

^a^ p < 0,05

^b^ Availability measures considered the availability of tracer medicines checked by the observation script, excluding items 38 to 45.

Source: PNAUM – Services, 2015.

The availability index ranged according to the population stratum (capitals, biggest cities of each region and smallest cities), and the lowest availability was registered in the smallest cities ( Figure 1). This variation occurred independently of the exclusion of phytotherapic medicines, with statistical significance (p<0.05).

**Figure:**
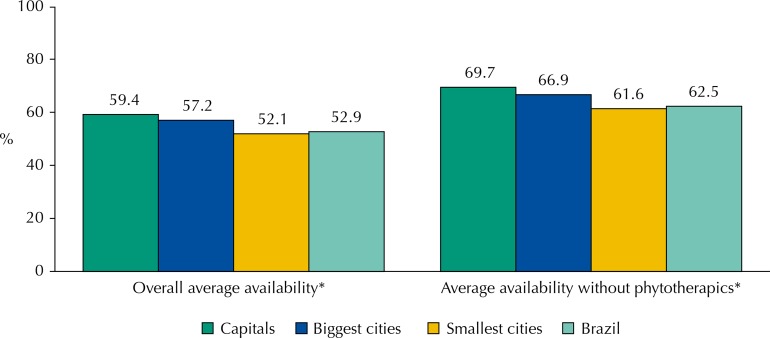
Average availability of tracer medicines in primary health care units, according to sampling stratum. National Survey on Access, Use and Promotion of Rational Use of Medicines – Services, 2015.

Source: PNAUM – Services, 2015.

* p < 0,05

Availability in the past three months was assessed from the perspective of patients, physicians, and medicine dispensers ( Table 3 ). According to 58.8% of physicians, the availability of essential medicines was considered very good/good. When there is lack of medicines, the most frequent action is analyzing the possibility of replacing the medicine prescribed for therapeutic alternatives (77.4%), followed by referral to the Popular Pharmacy Program (75.4%).

**Table 3:** Perception of patients, physicians, and medicine dispensers in primary health care services on the availability of medicines. National Survey on Access, Use and Promotion of Rational Use of Medicines – Services, 2015.

	Perceived availability	n ^a^	% (IC95%)
Patients	Frequency of acquisition of needed medicines in the past three months ^b^		
	Always	3,357	59.8 (55.1–64.4)
	Repeatedly	410	7.1 (4.7–10.7)
	Sometimes	1,313	23.1 (20.0–26.5)
	Rarely	421	5.6 (4.6–6.9)
	Never	257	4.3 (3.0–6.1)
	Information received when did not receive the medicine(s) ^b^		
	Lack in unit	1,043	10.9 (8.7–13.5)
	Referred to another unit of the Brazilian Unified Health System	242	1.8 (1.2–2.8)
	Received instructions to buy the medicine	623	8.3 (6.7–10.1)
	Referred to the Popular Pharmacy Program	278	3.1 (2.4–4.0)
	Others	33	0.3 (0.2–0.4)
	No instructions	6,584	75.6 (72.5–78.5)
Physicians	Availability of medicines in the past three months		
	Very good/Good	654	58.8 (54.8–62.7)
	Neither bad/Nor good	437	34.3 (30.6–38.2)
	Bad/Very bad	95	6.9 (5.1–9.3)
	Conduct in situations of lack of medicines in the municipal health system ^b^		
	Analyses the possibility of replacing the medicine prescription	1,240	77.4 (74.2–80.4)
	Refers to the Popular Pharmacy Program in the city	1,202	75.4 (72.1–78.3)
	Recommends the purchase	1,090	69.3 (66.0–72.5)
	Refers to Aqui tem Farmácia Popular	1,049	66.8 (63.4–70.1)
Medicine dispensers	Lack of medicines in the past three months ^b^		
	Always	376	27.7 (21.8–34.5)
	Repeatedly	117	10.3 (6.2–16.6)
	Sometimes	412	35.6 (29.3–42.5)
	Rarely	141	16.3 (11.8–2.1)
	Never	91	10.1 (6.0–16.3)

^a^ Non-weighted n value

^b^ p <0,05

Source: PNAUM – Services, 2015.

Among the medicine dispensers, 38.0% reported lack of medicines takes place always or repeatedly in SUS primary health care units. According to those responsible for pharmaceutical services in the cities, insufficient financial resources (31.4%), problems in the pharmaceutical market (30.5%), delay in the transfer of medicines by other management instances of the SUS (27.2%), and disorganization of the local acquisition sector (18.8%) were the main reasons for shortages in the year before the survey (data not presented in table).

Among patients, 67.0% reported they always or repeatedly obtained the medicines they needed in SUS primary health care pharmacies. When the prescribed medicines were not provided, patients almost always did not receive further instructions from the dispensing unit (75.6%). In 10.9% of the cases, the lack of medicines was reported.

## DISCUSSION

The analysis of essential medicines’ availability is an important strategy to assess the impact of policies introduced in public health ^25^ . In Brazil, the importance of studies on access and quality of pharmaceutical services in the SUS is even greater, since there is evidence that the public supply is the only way to access medicines for low income families ^3^ ^,^ ^10^ .

This study found an average availability of 52.9%, which was higher than an average availability of 44.9% found by Mendes et al. ^13^ for essential medicines in Brazilian primary health care, and similar to the average of 40 developing countries found by Cameron et al. ^7^

According to the regulation of the Basic Component of Pharmaceutical Services (CBAF) in the SUS ^c^ , cities can create a list of medicines that meet local demand, since the items are covered by the ruling Rename Appendices. However, as observed by Helfer et al. ^10^ , this autonomy cannot prevent professionals from meeting the prevalent injuries. One cannot affirm that the low general availability has been counterbalanced by the provision of locally standardized alternatives ^3^ ^,^ ^10^ ^,^ ^13^ . A limitation of data analysis presented in this study is the lack of access to the respective municipal lists.

General availability varied according to the regions of the Country, being lower in the North and Northeast regions (44.6% and 46.3%, respectively). These regions also showed the lowest indexes for the following variables: existence of a responsible pharmacist, exclusive refrigerator for medicines, and locker cabinets for medicines under special control. These data corroborate Mendes et al. ^13^ findings, which associated higher availability of medicines in primary health care with adequate infrastructure (storage area, air conditioning, and refrigerator) and pharmacist support.

Availability varied according to the population size – capitals and the 0.5% largest cities included in the sample presented better availability than other cities. This relationship between improved availability and size of population was also identified in Brazil by other studies in which medicine supply rates were higher in bigger cities ^3^ ^,^ ^13^ . These findings suggest structural and management deficiencies in small cities to properly meet local health demands.

Overall average availability, when excluding phytotherapic medicines, was 62.5%, similar to Mendes et al. ^13^ findings (58.5%). Although the national policy and funding provided by CBAF ^19^ , phytotherapic medicines were available in only 8.8% of the units. The low availability of these medicines shows the need for using strategies for rendering effective integrative and complementary practices in the Country, to ensure the adoption of innovative and socially contributory alternatives. Brazil has many advantages and opportunities for the development of this therapy, such as the world’s greatest plant diversity, huge socio-diversity, and traditional knowledge ^6^ ^,^ ^20^ .

Low availability of medicines in the public sector is a global issue, and medicines for chronic diseases are even scarcer than those for acute diseases, particularly in low and middle income countries ^7^ . Medicines are very important in treatment of morbidities that present increasing prevalence in Brazil due to population ageing ^3^ ^,^ ^12^ , such as chronic degenerative diseases (hypertension and diabetes mellitus) and mental health problems.

In this study, indexes above the 80% recommended by the WHO ^3^ ^,^ ^25^ were found for some antihypertensive medicines (captopril/enalapril and hydrochlorothiazide) and oral antidiabetic medicines (metformin, glyburide/gliclazida), but they were lower for beta-blockers, insulin, psychotropic medicines, and those for asthma treatment. These findings are consistent with some studies developed in the Country ^3^ ^,^ ^10^ ^,^ ^13^ .

Among the medicines for CNCD treatment, psychotropics presented the lowest availability. Although some studies ^10^ ^,^ ^13^ suggest a centralized supply of medicines subjected to special control in municipal public services, the low availability verified is relevant and represents a challenge to health care integrality. Treatment interruption as a result of shortages may result in hospitalizations for mental illness, reduce the patients quality of life, and increase health care costs. The improvement of access to essential psychotropic drugs is a key component to strengthen mental health care.

Infrastructure problems in SUS dispensing units can also be associated with the low availability verified for some items. Only 47.2% of the units had an exclusive refrigerator for storage of thermolabile medicines. Even though this index is higher than the 25.0% identified by Mendes et al. ^13^ , the low availability of NPH and regular human insulin (68.4% and 63.1%, respectively) is also found in many national and international studies ^3^ ^,^ ^4^ ^,^ ^13^ . Insulins are essential medicines to people with diabetes and are considered essential medicines the by WHO Global Action Plan ^26^ for the prevention and control of CNCD, and should always be available to the population.

Besides insulins, the Brazilian Ministry of Health purchases contraceptives and hormones for the Women Health Program, tuberculostatics, and smoking cessation medicines centrally. All of them presented availability below 80% in the national average, especially strategic medicines (tuberculostatics and nicotine), with availability inferior to 40% in all Country regions.

Tuberculosis is an important public health problem in Brazil, being the ninth cause of hospitalization and the fourth cause of mortality due to infectious diseases ^18^ . Ensuring access to medicines is an essential component in the reduction of the tuberculosis prevalence in the Country and, consequently, in the Americas. Thus, the low availability verified (22.9%) in primary health care units presents a challenge for the health system.

Low availability of medicines acquired centrally suggests eventual deficiencies in medicine supply chain. This hypothesis is consistent with the perception of professionals responsible for pharmaceutical services, who highlighted the delay in the transfer by the management instances of SUS as one of the main reasons for medicine shortage in the cities.

Regarding medicines for treatment of acute conditions, it is important to highlight the low availability of antiemetics and some anti-infective agents, such as benzathine benzylpenicillin. Eliminating congenital syphilis as a public health problem is a priority in Latin America and in the Caribbean region. WHO estimates one million cases of syphilis in pregnant women per year and preconize early detection and proper treatment of women and their partners to prevent serious implications for the baby ^5^ . Penicillin is the chosen treatment for cases of syphilis, and a continuous provision of this medicine is essential in primary health care services.

The different perceptions on availability of tracer medicines in the SUS corroborate the indexes of overall availability found in this study. Among patients, approximately 60% say they obtain the medicines needed in SUS units, data consistent with the lack of medicines reported by medicine dispensers and with the evaluation of primary health care physicians.

When there is lack of medicines, the most frequent actions described by the physicians were analyzing the possibility of replacing the medicine prescribed and referring the patient to the Popular Pharmacy Program. It is important to highlight that the first strategy is more feasible for the treatment of acute conditions. Replacement of continuous-use medicines may compromise the control of the disease and/or the adherence to therapy, affecting the treatment effectiveness. Regarding the referral to the Popular Pharmacy Program, only nine (18%) of the 50 checked items are provided for free ^d^ by private pharmacies participating in the program *Aqui Tem Farmácia Popular* or by the program distribution system. According to Helfer et al. ^10^ , the lack of access to medicines provided for free, especially medicines for CNCD, may compromise family budget and the treatment persistence, worsening the health status and consequently increasing expenses with outpatient visits and hospitalizations. Moreover, the majority of population that uses the public health system has low income, and free delivery is usually the only alternative of access to medicines.

Some limitations of this study must be considered. Availability was determined using a specific list of medicines, with a single data collection at each place. Therefore, availability can be under or overestimated, being impossible to evaluate the existence of an association between availability and local arrangements adopted for the medicines supply. Another important limitation relates to municipal lists of essential medicines. We did not evaluate the lists adopted by each city and, consequently, we were not able to identify the existence of therapy options for unavailable items. In addition, we considered as available the medicine with at least one posologic unit in stock, but would be important information about the existence of enough units for provision of therapeutic schemes, according to the demand of services ^10^ . Another limitation concerns the study design, which is cross-sectional and assesses availability in a determined period, and therefore does not allow concluding whether the lack or existence of medicines is constant or limited to that determined period.

Despite its limitations, this study presents important information about the availability of medicines in the public sector, mentioning issues in the implementation of medicines and pharmaceutical services policies, as well as the need for improving management and reorganization of pharmaceutical services to ensure the adequate supply of essential medicines. Frequent shortages of items considered essential may compromise the credibility of the public system ^4^ and affect health care costs. Availability of medicines for chronic diseases in developing countries is critical for treatment effectiveness and equity of access to these products ^24^ ^,^ ^25^ .

Although Brazil pioneered the adoption of a essential medicines list and of the public funding guaranteed by the SUS, the average availability of tracer medicines in all regions of the Country was lower than 80%, which is the percentage recommended by WHO. Therefore, the challenge of ensuring access to medicines in primary health care still remains. Low availability may affect treatments, raise health care costs, and impair the population quality of life.

The lowest availability rates identified in small cities, associated with the low availability of medicines purchased by the Brazilian Ministry of Health, shows eventual deficiencies in the supply chain and in the management of pharmaceutical services. Similar to the *Estratégia Saúde da Família* (FHS – Family Health Strategy), both the federal and state governments, along with cities, must assume the responsibility of providing minimum standards of structure and quality to dispensing units, to increase the availability of medicines in the SUS.

The evaluation of the essential medicines’ availability must be continuous, as part of a process of monitoring and evaluation of the medicines and pharmaceutical services’ national policies. The data here presented provide support for improving public pharmaceutical services and constitute a baseline to evaluate the long-term impact of strategies and policies adopted in the Country, aiming to improve the quality of services provided, health indicators, and thus ensure the population’s right to health.

## Footnotes

a The national policies of medicines and pharmaceutical services establish the guidelines for pharmaceutical services in Brazil.

b To make a research on the availability of essential medicines in health services feasible, the WHO suggests choosing a maximum of 50 items from a list of essential medicines. This list of key medicines, called tracer medicines, must contain items destined to treatment of main health conditions of the population, including a standard list of 14 medicines, to allow international comparisons, a regional basic list of up to 16 medicines, and a complementary list of at least 20 medicines of national relevance (WHO, 2008).

c Rules for funding and implementation of the Basic Component of Pharmaceutical Assistance within the Brazilian Unified Health System are regulated by governmental decree no. 1,555 from July 30, 2013.

d Medicines provided freely by the Popular Pharmacy Program comprise the strategy *Saúde não tem preço* , which includes medicines for hypertension, diabetes, and asthma. Ministério da Saúde (BR), Elenco oficial dos medicamentos disponibilizados gratuitamente pelo Programa Aqui tem Farmácia Popular (updated in 01/29/2016). Brasília (DF); 2016 [cited 2016 March 4]. Available from: http://portalsaude.saude.gov.br/images /pdf/2016/fevereiro/02/rol-medicamentos-SNTP-290116.pdf
